# Genetic Diversity and Positive Selection Analysis of Classical Swine Fever Virus Envelope Protein Gene E2 in East China under C-Strain Vaccination

**DOI:** 10.3389/fmicb.2016.00085

**Published:** 2016-02-05

**Authors:** Dongfang Hu, Lin Lv, Jinyuan Gu, Tongyu Chen, Yihong Xiao, Sidang Liu

**Affiliations:** Department of Animal Science and Technology, Shandong Agricultural UniversityTai’an, China

**Keywords:** classical swine fever virus, genetic diversity, phylogenetic analysis, positive selection, subgenotype 2.1d

## Abstract

Classical swine fever virus (CSFV) causes an economically important and highly contagious disease of pigs worldwide. C-strain vaccination is one of the most effective ways to contain this disease. Since 2014, sporadic CSF outbreaks have been occurring in some C-strain vaccinated provinces of China. To decipher the disease etiology, 25 CSFV E2 genes from 169 clinical samples were cloned and sequenced. Phylogenetic analyses revealed that all 25 isolates belonged to subgenotype 2.1. Twenty-three of the 25 isolates were clustered in a newly defined subgenotype, 2.1d, and shared some consistent molecular characteristics. To determine whether the complete E2 gene was under positive selection pressure, we used a site-by-site analysis to identify specific codons that underwent evolutionary selection, and seven positively selected codons were found. Three positively selected sites (amino acids 17, 34, and 72) were identified in antigenicity-relevant domains B/C of the amino-terminal half of the E2 protein. In addition, another positively selected site (amino acid 200) exhibited a polarity change from hydrophilic to hydrophobic, which may change the antigenicity and virulence of CSFV. The results indicate that the circulating CSFV strains in Shandong province were mostly clustered in subgenotype 2.1d. Moreover, the identification of these positively selected sites could help to reveal molecular determinants of virulence or pathogenesis, and to clarify the driving force of CSFV evolution in East China.

## Introduction

Classical swine fever (CSF), previously known as hog cholera, is an economically important, highly contagious disease of pigs that is classified as a notifiable disease by the Office International des Epizooties (Jiang et al., 2013). CSF is characterized by fever and hemorrhage with an acute or chronic course (Luo et al., 2011). CSF was first recognized in Tennessee, USA, in 1810, and then rapidly spread throughout the world (Edwards et al., 2000). As a result of systemic immunizations with live attenuated vaccines and/or strict epidemiological surveillance, CSF had been controlled and successfully eradicated from domestic pigs in some countries and regions, such as Australia, New Zealand, North America, and Western Europe (Paton and Greiser-Wilke, 2003; Ji et al., 2015). However, it still significantly affects swine production in Asia, South America, Eastern Europe, and parts of the former Soviet Union (Ji et al., 2015).

The causative agent, CSFV, is a member of the genus *Pestivirus* within the family *Flaviviridae* (Lowings et al., 1996). The positive-sense, single-stranded RNA CSFV genome is 12.3 kb in length, and it comprises one large ORF that is flanked by two NTRs (Rumenapf et al., 1991; Tautz et al., 2015). The ORF codes a 3898-AA polyprotein that is co- and post-translationally processed by cellular and viral proteases into four structural (C, Erns, E1, and E2) and eight non-structural proteins in the order NH2–(Npro-C-Erns-E1-E2-p7-NS2-NS3-NS4A-NS4B-NS5A-NS5B)–COOH (Rumenapf et al., 1991; Chang et al., 2010). The E2 protein is the main immunogen of CSFV, and it induces the production of neutralizing antibodies that provide protection against lethal challenge (Beer et al., 2015); it also plays multiple roles in the viral life cycle, and it mediates the entry of the virus into host cells (Sanchez et al., 2008; Shen et al., 2011).

The various isolates of CSFV consist of one serotype, reflecting a narrow range of evolutionary divergence (Vanderhallen et al., 1999; Deng et al., 2005). Therefore, genetic typing of the virus has been used to understand the evolution and spread of viruses, and the origins of disease outbreaks (Deng et al., 2005). 5′-NTR (96 nt), partial E2 (190 nt), and NS5B (409 nt) sequence similarities are extensively used for genetic analyses and to study viral diversity (Lowings et al., 1996; Greiser-Wilke et al., 1998; Paton et al., 2000). Recently, the full-length E2 coding sequence (1,119 nt) was also demonstrated to be reliable in detailed phylogenetic analyses (Postel et al., 2012; Beer et al., 2015). Analyses using these three or four regions have similarly classified CSFV into three genotypes, each with three to four subgenotypes (Lowings et al., 1996; Paton et al., 2000; Deng et al., 2005). Thus far, subgenotypes 1.1–1.4, 2.1–2.3, and 3.1–3.4 can be differentiated (Lowings et al., 1996; Paton et al., 2000; Postel et al., 2013).

Determining the selection pressures that have shaped the genetic variation of viruses is a major part of many molecular evolution studies (Kosakovsky Pond and Frost, 2005). A powerful method for studying adaptive molecular evolution is the use of a codon substitution model to identify AA sites where the dN exceeds the dS in a maximum likelihood context (Anisimova et al., 2001; Shen et al., 2011). Estimates of dN that are significantly different from dS provide convincing evidence for non-neutral evolution (Kosakovsky Pond and Frost, 2005). In viruses, the AAs at the interacting sites between envelope proteins and host molecules are continuously evolving under positive selection (Shen et al., 2011).

Since late 2014 in many regions of Shandong province in East China, a CSF epidemic, which is characterized by abortions and stillbirths of sows, as well as fever, anorexia, skin hemorrhages, and high-mortality among nursery pigs, has been occurring in many pig herds that were immunized with attenuated CSFV vaccines (the C strain, Hog Cholera Lapinized Virus). Most pigs in Shandong are vaccinated according to the following schemes: sows and boars are vaccinated simultaneously three times per year. Piglets are vaccinated first via an intramuscular injection at 21–28 days of age, and they receive a second vaccination at 7–8 weeks of age. Replacement gilts and boars are then vaccinated at 12–16 weeks, followed by a supplementary immunization before estrus (unpublished data). Here, we conducted a molecular epidemiological survey of 25 CSFV isolates and showed that the circulating CSFV strains in Shandong province were mostly clustered in subgenotype 2.1d. The selection pressures that act on the E2 gene of these new isolates and 120 reference strains were further analyzed to obtain insights into the driving forces of CSFV evolution in swine populations under regular vaccination programs.

## Materials and Methods

### Sample Preparation and Virus Isolation

A total of 169 tissue specimens, including the spleen, lymph nodes, tonsils, brain, lungs, and kidneys, were collected from clinically ill nursery pigs from different pig herds of various sizes in Shandong province from December 2013 to June 2015. The tissue samples were collected in accordance with the guidelines of the Shandong Agricultural University Animal Care and Use Committee (SDAUA-2013-001) and dissected for cryopreservation and fixed in 10% neutral formalin for virus detection and histological examination, respectively. Tissue samples were homogenized in Dulbecco’s modified Eagle’s medium (Gibco, Grand Island, NY, USA), and then the tissue homogenates were centrifuged at 10,000 × *g* (4°C) for 10 min. Then, the suspension was passed through a 0.22-μm filter (EMD Millipore, Billerica, MA, USA) and transferred to PK-15 cell monolayers. Then, the cells were incubated at 37°C in 5% CO_2_ for 3–5 days, and the cultures were harvested and stored at –80°C as viral stocks.

### Histological Examination and Polymerase Chain Reaction (PCR) Detection

The formalin-fixed samples were processed and embedded in paraffin. Thin sections of the fixed tissues were stained with H&E and examined microscopically. Viral DNA and RNA of the harvested cultures were extracted using the EasyPure viral DNA/RNA kit (TransGen, Beijing, China) according to the manufacturer’s instructions for the detection of suspected viruses. Four major pathogens, including CSFV, PRRSV, PRV, and PCV2 were detected by PCR or reverse transcription (RT)-PCR (Hu et al., 2015).

### E2 Gene Amplification and Sequencing

Primers based on the published sequence of the CSFV Shimen strain (GenBank accession no. AF092448) were designed to amplify the complete E2 gene (forward primer: GTAAATATGTGTGTGTTAGACCAGA, reverse primer: GTGTGGGTAATTRAGTTCCCTATCA; Zhang et al., 2015). The viral RNA of CSFV-positive cultures was extracted, and the complete E2 gene was amplified using the EasyScript One-Step RT-PCR SuperMix (TransGen, Beijing, China). Briefly, 6 μL of RNA template, 25 μL of Reaction Mix, 1 μL of Enzyme Mix, and 16 μL of RNase-free water were mixed with 1 μL of each primer (10 μM). One-step RT-PCR was performed using the following conditions: 45°C for 25 min, 94°C for 5 min, followed by 30 cycles of 94°C for 30 s, 55°C for 30 s, and 72°C for 2 min, followed by a final extension at 72°C for 7 min. PCR/RT-PCR products were analyzed by 1% agarose gel electrophoresis. Target fragments were excised from the gels for purification using the Gel Extraction Kit (Tiangen, Beijing, China). Purified PCR products were cloned into the pMD18-T vector (TaKaRa, Beijing, China). Recombinant clones and the forward and reverse primers were sent to Sangon Bioscience (Shanghai, China) for sequencing.

### Phylogenetic Analysis of the E2 Gene

The E2 gene sequences that were amplified from the clinical samples (**Table 1**) were aligned with 120 sequences in GenBank (Supplementary Table S1), and phylogenetic trees were constructed using MEGA 6.0 software^1^ by the maximum likelihood method based on the Tamura–Nei model (Tamura and Nei, 1993; Tamura et al., 2013). Bootstrap values were estimated for 1,000 replicates. Trees were determined based on the full-length E2 sequence (1,119 nt) and a partial E2 sequence (190 nt) (Lowings et al., 1996).

**Table 1:** Characteristics of the 25 new isolates and GenBank accession no. of E2 genes (1,119 nt).

No.	Isolates name	Specimen	Time	Accession no.
1	SDJNi1-15	Spleen, lymph nodes	2015.04.25	KT953587
2	SDJNi2-15	Brain, tonsil, lung	2015.03.13	KT953588
3	SDJNa-14	Brain, tonsil, lung	2014.04.12	KT953589
4	SDZB2-15	Spleen, lymph nodes	2015.03.20	KT953590
5	SDTA2-15	Tonsil, lung	2015.04.09	KT953591
6	SDTA1-13	Spleen, lymph nodes, kidneys	2013.12.25	KT953592
7	SDLW1-15	Tonsil, lung	2015.04.01	KT953593
8	SDLY-15	Spleen, tonsil, lung	2015.04.07	KT953594
9	SDTA3-15	Tonsil, lung	2015.04.09	KT953595
10	SDJNi3-15	Spleen, lymph nodes	2015.03.06	KT953596
11	SDLW2-15	Tonsil, lung	2015.03.05	KT953597
12	SDJNi4-15	Tonsil, lung	2015.04.29	KT953598
13	SDMZ1-15	Spleen, lymph nodes	2015.04.25	KT953599
14	SDJNi5-15	Brain, tonsil, lung	2015.04.25	KT953600
15	SDJNi6-15	Tonsil, lung	2015.04.25	KT953601
16	SDWK-15	Tonsil, lung	2015.04.15	KT953602
17	SD19-15	Spleen, lymph nodes	2015.03.11	KT953603
18	SDLY-14	Spleen, lymph nodes, tonsil	2014.12.30	KT953604
19	SDZB-15	Tonsil, brain, kidneys	2015.06.03	KT953605
20	SDMZ2-15	Spleen, lymph nodes	2015.06.09	KT953606
21	SDHZ-15	Tonsil, lung	2015.06.08	KT953607
22	SDSK-15	Spleen, lymph nodes	2015.06.08	KT953608
23	SDXLS-15	Spleen, lymph nodes	2015.06.15	KT953609
24	SDTA4-15	Brain, tonsil, lung	2015.06.10	KT953610
25	SDXT-15	Spleen, lymph nodes, tonsil	2015.06.03	KT953611

### Identities and AA Substitution Analysis of the E2 Gene/Protein

The nt and AA sequence identities of the 25 new CSFV isolates and eight representative CSFV isolates, including Shimen (AF092448, 1.1), SXCDK (GQ923951, 2.1a), HEBZ (GU592790, 2.1b), GDPY2008 (HQ697223, 2.1c), SDQS (JQ001834, 2.1d), LAL290 (KC851953, 2.2), Novska (HQ148061, 2.3), and TWN (AY646427, 3.4), were calculated using the MegAlign module (Clustal W method) of the Lasergene package (DNASTAR Inc., Madison, WI, USA). The AA substitutions of the new isolates were compared with those of the representative CSFV isolates, which included three genotypes (1.1–1.4, 2.1–2.3, and 3.4).

### Selection Pressure Analysis of the E2 Gene

An analysis of the selection pressure acting on the codons of the E2 envelope protein, including the 25 new isolates and 120 reference strains, was conducted using the HyPhy open-source software package available at the datamonkey web-server^2^ (Delport et al., 2010). The level of positive selection was estimated using five different approaches: single likelihood ancestor counting (SLAC), fixed effects likelihood (FEL), internal fixed effects likelihood (IFEL), mixed effects model of evolution (MEME), and fast unbiased Bayesian approximation (FUBAR) (Sharma et al., 2013). The best nucleotide substitution model for different datasets, as determined via the available tool on the datamonkey server, was used in the analysis.

## Results

### Gross and Histological Lesions of CSF-Suspected Cases

**FIGURE 1:**
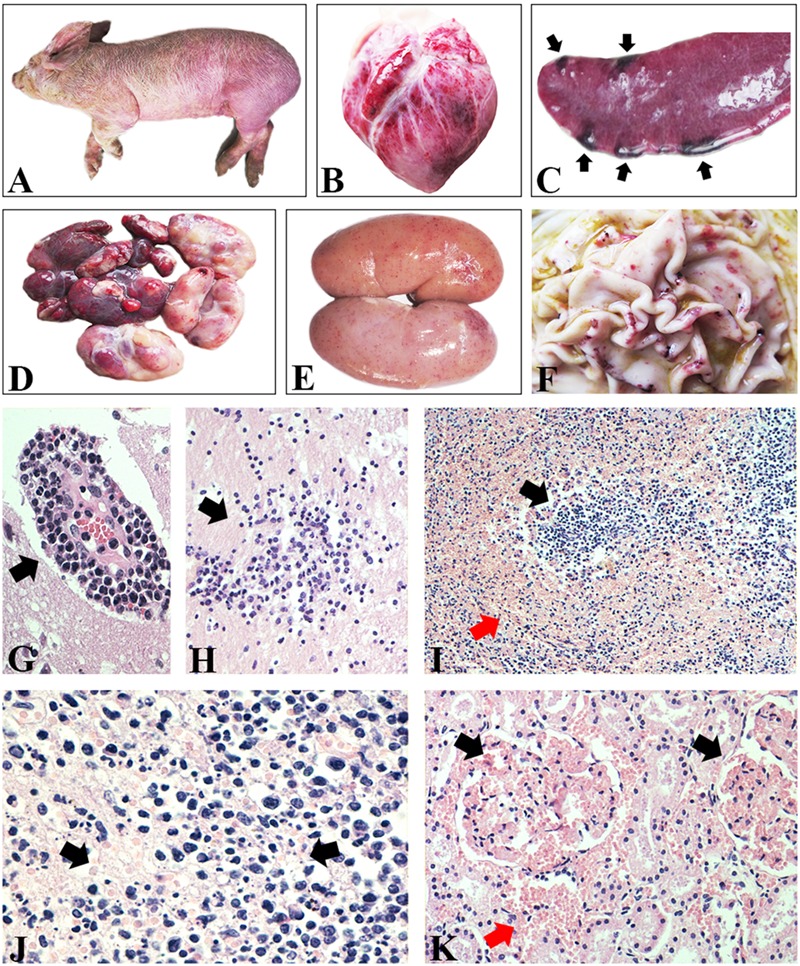
**Gross and histological lesions of CSF-suspected pigs. (A)** Diffuse hemorrhage of skin. **(B)** Epicardium hemorrhage. **(C)** Infarcts scattered on the edge of spleen. **(D)** Lymph nodes were hemorrhagic and turgid. **(E)** Renal cortex was densely covered with petechial hemorrhages. **(F)** Hemorrhagic spots and ulcer on the surface of gastric mucosa. **(G)** Lymphocyte infiltration around the small blood vessels in brain. H&E stain, ×400. **(H)** Proliferation of glial cells in brain. H&E stain, ×200. **(I)** Histological structure of spleen was disordered and characterized by necrotic lymphocytes and hemorrhage. H&E stain, ×100. **(J)** Lymphoid nodules showed necrotic lymphocytes and hyperplastic reticular cells and hemorrhage. H&E stain, ×400. **(K)** The glomerulus and mesenchyme were hemorrhagic. H&E stain, ×200.

Systematic necropsies were performed on pigs with clinical signs of CSF, including fever, anorexia, diffuse hemorrhage of the skin (**Figure 1A**), and conjunctivitis. Obvious hemorrhagic spots were found on the surface of the epicardium (**Figure 1B**). Scattered hemorrhagic infarcts were observed on the edge of the spleen (**Figure 1C**). Multiple lymph nodes were hemorrhagic and turgid (**Figure 1D**). The renal cortex were densely covered with petechial hemorrhages (**Figure 1E**). A mixture of small and large hemorrhagic spots, as well as ulcers, was seen on the surface of the gastric mucosa (**Figure 1F**). Histological examination mainly confirmed viral encephalitis, hemorrhages of many tissues, and necrotic foci of lymphoid tissues. The brain tissue exhibited typical viral encephalitis with lymphocyte infiltration around the small blood vessels (**Figure 1G**), as well as the proliferation of glial cells (**Figure 1H**). The histological structure of the spleen was disordered and characterized by necrosis, hemorrhage, and depletion of lymphocytes (**Figure 1I**). The lymph nodes showed hemorrhagic necrotizing lymphadenitis with necrotic lymphocytes and hyperplastic reticular cells (**Figure 1J**). The glomerulus and mesenchyme were hemorrhagic (**Figure 1K**).

### Pathogens Detected in the Clinical Samples

The PCR/RT-PCR results showed that 25 of the 169 tissue specimens collected from different herds were positive for CSFV. Among the 25 samples, 12 samples were positive for PCV2, five for PRV, and four for PRRSV (data not shown). All 25 amplified E2 genes were sequenced and submitted to GenBank (**Table 1**).

### Phylogenetic Analysis of the E2 Gene

**FIGURE 2:**
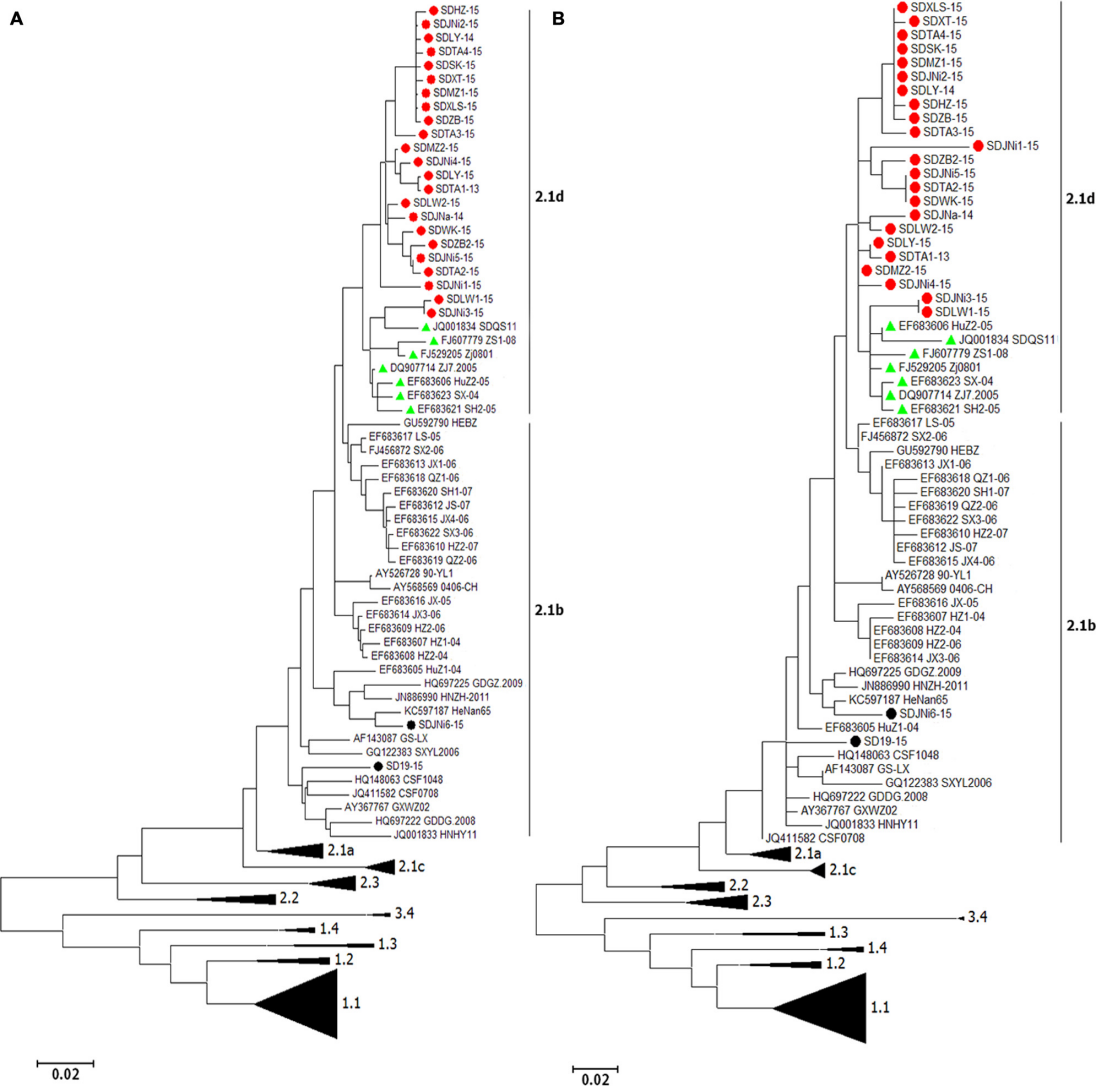
**Phylogenetic analysis of different genomic regions of the 25 new isolates and 122 reference strains. (A)** Full-length E2 gene sequences. **(B)** Partial E2 gene sequences. 23 newly isolated strains (indicated by 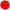) accompanied with seven references strains (indicated by 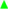) were clustered into subgenotype 2.1d. The other two newly isolated strains (labeled by 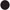) belonged to subgenotype 2.1b.

A total of 145 full-length E2 gene (1,119 nt) sequences and 145 corresponding partial E2 gene (190 nt) sequences, including the sequences of the 25 new isolates, were used to construct phylogenetic trees (**Figure 2**). The analysis resulted in a classification of all 145 CSFVs into three main groups (genotypes 1–3) containing eight subgroups (1.1–1.4, 2.1–2.3, and 3.4; **Figure 2**).

Of the 25 new isolates, 21 isolates that were isolated in 2015 (SDHZ-15, SDJNi2-15, SDLY-15, SDTA4-15, SDSK-15, SDXT-15, SDMZ1-15, SDXLS-15, SDZB-15, SDTA3-15, SDMZ2-15, SDJNi4-15, SDLY-15, SDLW2-15, SDWK-15, SDZB2-15, SDJNi5-15, SDTA2-15, SDJNi1-15, SDLW1-15, and SDJNi3-15), two previously isolated strains (SDTA1-13 in 2013 and SDJNa-14 in 2014), and seven previously sequenced isolates [SDQS11 (JQ001834), ZS1-08 (FJ607779), Zj0801 (FJ529205), ZJ7.2005 (DQ907714), HuZ2-05 (EF683606), SX-04 (EF683623), and SH2-05(EF683621)] belonged to the new subgenotype 2.1d (Zhang et al., 2015). The remaining two new isolates, SD19-15 and SDJNi6-15, were clustered in subgenotype 2.1b. Phylogenetic trees based on the two different gene sequences, including the 145 full-length E2 gene sequences (**Figure 2A**) and 145 partial E2 gene sequences (**Figure 2B**), produced similar results. It is evident that all of the recently isolated CSFV strains in Shandong province were surprisingly divergent from the Shimen reference strain and the vaccine strain HCLV, and that the subgenotype 2.1 CSFV strains (mainly subgenotype 2.1d) predominated in more recent CSF epidemics in Shandong province in East China.

### Site Mutation Analysis of the E2 Gene

The E2 gene of the 25 new isolates is 1,119 nt long, encoding a 373-AA protein. When compared with each of the eight reference strains (**Table 2**), the 25 newly isolated strains shared the lowest nt identities (81.7–82.5%) and AA identities (88.5–90.1%) with the TWN strain (subgenotype 3.4). The new isolates shared the highest nt and AA sequence similarities with 2.1 reference strains. When compared with each of the four subgenotypes of genotype 2.1, the two new isolates, SD19-15 and SDJNi6-15, had the highest nt identity (94.2%) and AA identities (96.2 and 97.1%, respectively) with the 2.1b reference strain HEBZ, while the other 23 new isolates shared the highest nt identities (95.9–97.5%) and AA identities (96.2–98.4%) with the 2.1d reference strain SDQS (**Table 2**, Supplementary Tables S2 and S3). In addition, the 25 new isolates had greater similarities to subgenotype 2.1b isolates than to either subgenotype 2.1a or 2.1c isolates, indicating a high similarity between subgenotypes 2.1b and 2.1d; these results are in accordance with the report by Zhang et al. (2015).

**Table 2:** Nucleotide (nt) and AA identities of E2 gene between the 25 new isolates and other eight representative CSFV isolates (%).

Identities	Shimen (1.1)	SXCDK (2.1a)	HEBZ (2.1b)	GDPY2008 (2.1c)	SDQS (2.1d)	LAL290 (2.2)	Novska (2.3)	TWN (3.4)
nt	83.1–84.2	90.5–92.0	94.2–96.3	90.3–92.1	93.7–97.5	85.3–86.7	86.0–87.4	81.7–82.5
AA	88.7–90.9	93.3–96.0	95.4–97.6	94.1–96.8	95.7–98.4	89.5–92.0	90.9–93.6	88.5–90.1

**FIGURE 3:**
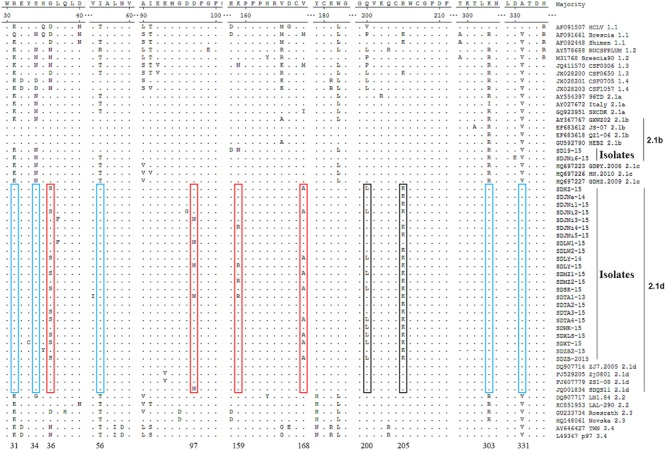
**Amino acid (AA) sequences alignments of E2 genes of the 25 new isolates and 29 representative CSFV isolates.** Consistent molecular characteristics of 2.1d strains were indicated by blue boxes 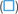. Unique AA substitutions of 2.1d strains compared with all other strains were indicated by red boxes 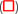. Unique AA substitutions of 2.1d strains compared with subgenotype 2.1a, 2.1b, and 2.1c strains were indicated by black boxes 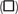.

Compared with the reference strains, the two 2.1b new isolates, SD19-15 and SDJNi6-15, showed no characteristic AA substitutions, while the other 23 new isolates, which belonged to the 2.1d subgenotype, had some unique characteristics (**Figure 3**). Compared with all of the other isolates, the new 2.1d isolates, as well as four of the 2.1d reference strains (DQ907714, FJ529205, FJ607779, and JQ001834) showed consistent AA substitutions, including an R at position 31 (R^31^), S^34^, I^56^, K^303^, and A^331^. The subgenotype 2.1d isolates also showed unique AA substitutions, including G/D/N^36^S, D^97^N, K/N^159^R, and V/M/I^168^A. In addition, some subgenotype 2.1d isolates had two AA substitutions at positions 200 (Q^200^L) and 205 (R^205^K) compared with subgenotype 2.1a, 2.1b, and 2.1c isolates.

### Selection Pressure Analysis

A selection pressure analysis of the E2 gene of 145 global CSFV strains revealed seven positively selected sites (AAs 17, 34, 72, 168, 200, 240, and 283) by at least two methods (**Table 3**). The detected positively selected sites were diverse, and most of the sites were hydrophilic sites (**Table 4**). There were no regular changes in polarity of the positively selected AAs, but a change from a polar AA (Q) at position 200 to non-polar AAs (V, P, and L) was observed (**Table 4**).

**Table 3:** Selection pressure analysis of E2 protein (373 codons) of CSFV using SLAC, FEL, IFEL, MEME, and FUBAR methods (www.datamonkey.org).

Protein	Codon^∗^	SLAC		FEL		IFEL		MEME		FUBAR	
		dN-dS	*p*	dN/dS	*p*	dN/dS	*p*	β^+^	*p*	β–α	*Post.Pr.*^†^
E2	17	None	–	None	–	+∞	0.032	None	–	0.118	0.749
	34	4.949	0.149	3.692	0.058	4.492	0.045	1.618	0.076	0.278	0.856
	72	8.142	0.010	+∞	0.004	+∞	0.005	27.412	0.001	0.524	0.971
	168	3.718	0.172	None	–	None	–	4.720	0.035	0.147	0.777
	200	5.768	0.132	None	–	None	–	None	–	0.437	0.820
	240	3.320	0.160	+∞	0.026	+∞	0.072	0.679	0.039	0.190	0.882
	283	2.759	0.198	+∞	0.063	+∞	0.089	218.761	0.020	0.124	0.806

*The sites found under positive selection (significance value) by at least two methods are shown.*

*Codon with *p* < 0.2 level in SLAC method, or with *p* < 0.1 level in FEL method, or with *p* < 0.1 level in IFEL method, or with *p* < 0.1 level in MEME method, or with *Post.Pr.* ≥ 0.7 level in FUBAR method, was considered under positive selection.*

*^∗^Positively selected sites (AA).*

*^†^Posterior probability.*

**Table 4:** Positions and polarities of the positively selected amino acid (AA).

Sites	17	34	72	168	200	240	283
Majority	E^-^	S^0^	R^+^	V^×^	Q^0^	R^+^	R^+^
Substitution	K^+^, G^0^	N^0^, D^-^, G^0^	G^0^, E^-^, D^-^	M^×^, A^×^, L^×^, I^×^	V^×^, P^×^, L^×^	G^0^, K^+^	C^-^, H^+^, Y^0^

*The polarities of positively selected AA were indicated at the top-right corner of each AA.*

*^×^Non-polar AA (hydrophobic).*

*^+^Positively charged AA (hydrophilic).*

*^-^Negatively charged AA (hydrophilic).*

*^0^Uncharged AA (hydrophilic).*

## Discussion

In China, a nationwide policy of biannual vaccinations of pigs in the spring and autumn has been performed using the C-strain vaccine, and large-scale outbreaks of CSF have rarely occurred since its introduction (Shen et al., 2011). Some of the cases that occurred were acute, but many cases of CSF were seen as subclinical, causing reproductive failure, neonatal death, or chronic infection in nursery pigs (Luo et al., 2011; Ji et al., 2015). However, in 2014, pigs in some herds in China that were immunized with attenuated CSFV vaccines showed CSF-suspected symptoms (Zhang et al., 2015), and subsequently a similar epidemic unexpectedly occurred in Shandong province, which caused heavy economic losses. To identify the pathogeneses and pathogens, specimens were collected and systemic examinations were performed, and the CSFV infection status was confirmed.

To further study the molecular epidemiology of CSF, 25 isolated CSFV strains were obtained, and their genetic diversity was analyzed. The full-length E2 gene sequence (1,119 nt), which provides better resolution for phylogenetic analysis than 5′-NTR, partial E2 gene, and NS5B sequences (Blacksell et al., 2004; Sarma et al., 2011; Zhang et al., 2015), was sequenced and examined in this study. Both the full-length E2 sequence and partial E2 sequence showed similar results, as the CSFV isolates could be divided into three genotypes (1, 2, and 3) as well as 11 subgenotypes [1.1–1.4, 2.1 (2.1a, 2.1b, 2.1c, and 2.1d), 2.2, 2.3, and 3.4]. Compared with representative strains of subgenotypes 1.1, 2.1, 2.2, 2.3, and 3.4, the 25 isolates all belonged to subgenotype 2.1, and most of the strains (92%, 23/25) were clustered in the newly defined subgenotype 2.1d (**Figure 2**, **Table 2**). High sequence variability is found in mainland China where CSFV subgenotype 1.1, 2.1, 2.2, and 2.3 strains are found, and subgenotype 2.1b has been shown to be the predominant strains within the last 10 years (Tu et al., 2001; Chen et al., 2010a; Beer et al., 2015). In this study, CSF cases caused by a new subgenotype, 2.1d, of CSFV in Shandong province were diagnosed following outbreaks in other provinces (Zhang et al., 2015), and the earliest discovered CSFV isolate, SDTA1-13, which was identified as subgenotype 2.1d in this study, was first isolated in 2013. The results indicate that the new strains may have emerged over a short period of time and spread to several provinces in China, which is worthy of attention because all of the new strains were isolated from CSFV-immunized pigs (Zhang et al., 2015). The pathogenicity, antigenicity, and virulence of the newly defined 2.1d isolates remain unclear, but we speculate that the unique molecular characteristics of the 2.1d isolates may contribute to the adaptive evolution of CSFV under C-strain vaccination, and may be responsible for the unsatisfactory immunoprotection of C-strain vaccinations.

To further study the molecular characteristics of CSFV strains, a selection pressure analysis of E2 AA sequences was performed, and the results showed that the protein mainly underwent purifying selection pressures. RNA viruses are known to have significantly greater mutation rates per site per round of replication than DNA viruses, a difference that is attributed to the error-prone nature of viral RNA-dependent RNA polymerases, and most mutations in coding regions are deleterious (Weiss, 2002; Hughes and Hughes, 2007). A mechanism to decrease the accumulation of deleterious mutations is essential for RNA viruses to remain stable, and purifying selection provides a useful tool to purge such mutations (Domingo and Holland, 1997). In addition, purifying selection was reportedly more effective in RNA viruses than in DNA viruses (Hughes and Hughes, 2007). Seven positively selected sites were observed in the E2 protein, which is the main immunogen of CSFV. E2 is a type I transmembrane protein with a transmembrane domain in its carboxyl-terminus that is anchored in the viral envelope (Li et al., 2013). The amino-terminal half of the E2 protein, which is an extracellular motif that contains four antigenic domains (A, B, C, and D), was more variable than the carboxyl-terminal half (van Rijn et al., 1994). E2 has a unique architecture consisting of two immunoglobulin-like domains (I and II). Domains D/A map to domain II (AAs 91–168) in the E2 crystal structure, and domains B/C correspond to domain I (AAs 1–90) (Li et al., 2013). Among the detected seven positively selected sites, AAs 17, 34, and 72 belonged to domains B/C. AA 168 belonged to domains D/A. Moreover, the other three sites (AAs 200, 240, and 283) are located in the carboxyl-terminal half of the E2 protein. Domains B/C, which form an independent antigenic unit, are responsible for antigenic specificity among various CSFVs, and the D/A domains of various CSFVs are relatively conserved (van Rijn et al., 1994; Chang et al., 2010). It has been reported that single mutations in the E2 B/C domains could lead to variations in viral neutralization (Chen et al., 2010b). The three positively selected sites found in domains B/C of the amino-terminal half of the E2 protein, which mediates viral entry into target cells, suggest that these changes could be associated with viral escape from neutralizing antibodies, and they could explain the lower severity of the clinical signs that developed in most of the affected animals. The positively selected AA 200 is reportedly necessary for the attenuation of the highly virulent Brescia strain, but the mechanisms mediating this attenuation remain unknown (Risatti et al., 2007; Tang et al., 2008). In this study, we observed a polarity change of AA 200 from hydrophilic to hydrophobic, which may contribute to a change of the antigenicity and virulence of CSFV. The other three positively selected sites (AAs 168, 240, and 283) found in this study are the first to be reported, and their biological significance needs to be further characterized. Understanding the functional importance of these positively selected AAs could help to predict possible changes in virulence, which will aid the study of the mechanism of immune evasion, and prevent CSF in the future.

## Conclusion

The 25 CSFV isolates from East China were clustered in subgroup 2.1, and most of the isolates, together with some previously sequenced strains, formed the newly defined subgenotype 2.1d, indicating that 2.1d CSFV strains may be predominant epidemic strains in Shandong province. The selection pressure analysis revealed that the envelope protein-encoding E2 gene had undergone positive selection, and several positively selected sites were identified, which could help to identify the molecular determinants of virulence or pathogenesis, and to clarify the driving force of CSFV evolution in East China. Empirical studies are required to assess the antigenicity and virulence of the 2.1d CSFV strains, as well as the influence of the positively selected AAs identified in this study on CFSV virulence or pathogenesis.

## Author Contributions

DH and SL contributed to conception and design of the study. YX contributed to design of the study. LL contributed to acquisition and analysis of data. JG and TC contributed to acquisition of data. DH and LL drafted the manuscript. YX and SL critically revised the manuscript.

## Conflict of Interest Statement

The authors declare that the research was conducted in the absence of any commercial or financial relationships that could be construed as a potential conflict of interest.

## Footnotes

1 http://www.megasoftware.net/

2 http://www.datamonkey.org/

## Abbreviations

AA: amino acid
CSFV: classical swine fever virus
dN: non-synonymous substitution rate
dS: synonymous rate
H&E: hematoxylin and eosin
nt: nucleotide
NTRs: non-translated regions
ORF: open reading frame
PCV2: porcine circovirus type 2
PRRSV: porcine reproductive and respiratory virus
PRV: porcine pseudorabies virus
