# Supplementary material for: Genetic Diversity and Positive Selection Analysis of Classical Swine Fever Virus Envelope Protein Gene E2 in East China under C-Strain Vaccination
Source: Front Microbiol. 2016 Feb 5;7:85. doi: 10.3389/fmicb.2016.00085 (PMC4742907; doi:10.3389/fmicb.2016.00085)
Supplement: Supplementary file 1 [file Table_1.DOC]

**Supplementary Table 1** Characteristics of the 120 reference CSFV strains.

| No. | Accession no. | Genotype | No. | Accession no. | Genotype | No. | Accession no. | Genotype |
| --- | --- | --- | --- | --- | --- | --- | --- | --- |
| 1 | AY568569 | 2.1b | 41 | EF683614 | 2.1b | 81 | HQ380239 | 1.1 |
| 2 | AF091507 | 1.1 | 42 | EF683615 | 2.1b | 82 | HQ380240 | 1.1 |
| 3 | AF091661 | 1.1 | 43 | EF683616 | 2.1b | 83 | HQ380241 | 1.1 |
| 4 | AF092448 | 1.1 | 44 | EF683617 | 2.1b | 84 | HQ380242 | 1.1 |
| 5 | AF099102 | 1.2 | 45 | EF683618 | 2.1b | 85 | HQ380243 | 1.1 |
| 6 | AF143087 | 2.1b | 46 | EF683619 | 2.1b | 86 | HQ380244 | 1.1 |
| 7 | AF326963 | 1.1 | 47 | EF683620 | 2.1b | 87 | HQ380245 | 1.1 |
| 8 | AF333000 | 1.1 | 48 | EF683621 | 2.1d | 88 | HQ697222 | 2.1b |
| 9 | AF352565 | 1.1 | 49 | EF683622 | 2.1b | 89 | HQ697223 | 2.1c |
| 10 | AF407339 | 2.2 | 50 | EF683623 | 2.1d | 90 | HQ697225 | 2.1b |
| 11 | AF531433 | 1.1 | 51 | EU490425 | 1.1 | 91 | HQ697226 | 2.1c |
| 12 | AY027672 | 2.1a | 52 | EU497410 | 1.1 | 92 | HQ697227 | 2.1c |
| 13 | AY072924 | 2.1a | 53 | EU789580 | 1.1 | 93 | HQ697228 | 2.1c |
| 14 | AY259122 | 1.1 | 54 | EU857642 | 1.1 | 94 | J04358 | 2.3 |
| 15 | AY367767 | 2.1b | 55 | EU915211 | 1.1 | 95 | JN886990 | 2.1b |
| 16 | AY382481 | 1.1 | 56 | FJ265020 | 2.3 | 96 | JQ001833 | 2.1b |
| 17 | AY526726 | 2.1a | 57 | FJ456872 | 2.1b | 97 | JQ001834 | 2.1d |
| 18 | AY526727 | 2.1a | 58 | FJ529205 | 2.1d | 98 | JQ411566 | 2.1a |
| 19 | AY526728 | 2.1b | 59 | FJ607779 | 2.1d | 99 | JQ411567 | 2.1a |
| 20 | AY526729 | 2.2 | 60 | GQ122383 | 2.1b | 100 | JQ411570 | 1.3 |
| 21 | AY554397 | 2.1a | 61 | GQ902941 | 2.1a | 101 | JQ411582 | 2.1b |
| 22 | AY578687 | 1.2 | 62 | GQ923951 | 2.1a | 102 | JX028200 | 1.3 |
| 23 | AY578688 | 1.2 | 63 | GU233731 | 2.3 | 103 | JX028201 | 1.4 |
| 24 | AY646427 | 3.4 | 64 | GU233732 | 2.3 | 104 | JX028203 | 1.4 |
| 25 | AY663656 | 1.1 | 65 | GU233733 | 2.3 | 105 | JX218094 | 2.1c |
| 26 | AY775178 | 1.1 | 66 | GU233734 | 2.3 | 106 | JX262391 | 2.1c |
| 27 | AY805221 | 1.1 | 67 | GU324242 | 2.3 | 107 | JX898523 | 2.1c |
| 28 | D49532 | 1.1 | 68 | GU592790 | 2.1b | 108 | JX898524 | 2.1c |
| 29 | D49533 | 1.1 | 69 | HM175885 | 1.1 | 109 | JX898525 | 2.1c |
| 30 | DQ127910 | 1.1 | 70 | HM237795 | 1.1 | 110 | KC503764 | 1.1 |
| 31 | DQ907714 | 2.1d | 71 | HQ148061 | 2.3 | 111 | KC533781 | 2.2 |
| 32 | DQ907717 | 2.2 | 72 | HQ148062 | 2.3 | 112 | KC533787 | 2.2 |
| 33 | EF683605 | 2.1b | 73 | HQ148063 | 2.1b | 113 | KC597187 | 2.1b |
| 34 | EF683606 | 2.1d | 74 | HQ380232 | 1.1 | 114 | KC851953 | 2.2 |
| 35 | EF683607 | 2.1b | 75 | HQ380233 | 1.1 | 115 | KM262189 | 1.1 |
| 36 | EF683608 | 2.1b | 76 | HQ380234 | 1.1 | 116 | L49347 | 3.4 |
| 37 | EF683609 | 2.1b | 77 | HQ380235 | 1.1 | 117 | M31768 | 1.2 |
| 38 | EF683610 | 2.1b | 78 | HQ380236 | 1.1 | 118 | U45477 | 1.1 |
| 39 | EF683612 | 2.1b | 79 | HQ380237 | 1.1 | 119 | U45478 | 1.1 |
| 40 | EF683613 | 2.1b | 80 | HQ380238 | 1.1 | 120 | U90951 | 1.1 |
