# Supplementary material for: Genetic Diversity and Positive Selection Analysis of Classical Swine Fever Virus Envelope Protein Gene E2 in East China under C-Strain Vaccination
Source: Front Microbiol. 2016 Feb 5;7:85. doi: 10.3389/fmicb.2016.00085 (PMC4742907; doi:10.3389/fmicb.2016.00085)
Supplement: Supplementary file 2 [file Table_2.DOC]

**Supplementary Table 2** Analysis of nucleotide (nt) identity of E2 gene between 25 new isolates and other 8 representative CSFV isolates (%).

| New isolates | Shimen  (1.1) | SXCDK  (2.1a) | HEBZ  (2.1b) | GDPY2008  (2.1c) | | SDQS  (2.1d) | LAL290  (2.2) | Novska  (2.3) | TWN  (3.4) |
| --- | --- | --- | --- | --- | --- | --- | --- | --- | --- |
| SD19-15 | 83.1 | 92.0 | **94.2** | 92.1 | 93.7 | | 86.3 | 87.4 | 82.3 |
| SDJNi6-15 | 83.7 | 91.8 | **94.2** | 91.0 | 93.8 | | 86.7 | 87.3 | 82.2 |
| SDHZ-15 | 83.1 | 90.5 | 95.6 | 90.3 | **95.9** | | 85.3 | 86.0 | 81.7 |
| SDJNa-14 | 83.6 | 91.3 | 96.1 | 90.8 | **96.6** | | 86.1 | 86.8 | 81.9 |
| SDJNi1-15 | 83.1 | 90.8 | 95.5 | 90.9 | **96.4** | | 86.3 | 86.3 | 82.0 |
| SDJNi2-15 | 83.4 | 90.8 | 95.8 | 90.5 | **96.2** | | 85.5 | 86.2 | 81.9 |
| SDJNi3-15 | 83.2 | 91.2 | 95.5 | 90.3 | **97.5** | | 86.0 | 87.0 | 82.1 |
| SDJNi4-15 | 83.4 | 91.1 | 95.9 | 90.6 | **96.4** | | 85.9 | 86.4 | 82.0 |
| SDJNi5-15 | 84.2 | 91.4 | 95.8 | 90.7 | **96.3** | | 86.0 | 86.7 | 82.2 |
| SDLW1-15 | 83.1 | 91.2 | 95.4 | 90.4 | **97.2** | | 85.9 | 87.0 | 82.2 |
| SDLW2-15 | 84.1 | 91.9 | 96.3 | 91.2 | **97.1** | | 86.1 | 86.7 | 82.2 |
| SDLY-14 | 83.3 | 90.7 | 95.7 | 90.4 | **96.1** | | 85.4 | 86.1 | 81.9 |
| SDLY-15 | 83.1 | 90.9 | 95.5 | 90.4 | **96.2** | | 85.5 | 86.6 | 81.8 |
| SDMZ1-15 | 83.4 | 90.8 | 95.8 | 90.5 | **96.2** | | 85.5 | 86.2 | 81.9 |
| SDMZ2-15 | 83.4 | 91.3 | 96.2 | 90.9 | **96.7** | | 86.0 | 86.7 | 82.2 |
| SDSK-15 | 83.3 | 90.7 | 95.7 | 90.4 | **96.1** | | 85.4 | 86.1 | 81.9 |
| SDTA1-13 | 83.1 | 90.9 | 95.5 | 90.4 | **96.2** | | 85.5 | 86.6 | 81.8 |
| SDTA2-15 | 84.0 | 91.2 | 95.6 | 90.5 | **96.2** | | 86.0 | 86.7 | 82.0 |
| SDTA3-15 | 83.4 | 91.0 | 96.1 | 90.5 | **96.4** | | 86.1 | 87.0 | 81.9 |
| SDTA4-15 | 83.2 | 90.6 | 95.6 | 90.3 | **96.0** | | 85.3 | 86.1 | 81.8 |
| SDWK-15 | 83.6 | 91.1 | 95.8 | 90.7 | **96.3** | | 85.5 | 86.5 | 82.0 |
| SDXLS-15 | 83.4 | 90.8 | 95.8 | 90.5 | **96.2** | | 85.5 | 86.2 | 82.1 |
| SDXT-15 | 83.3 | 90.6 | 95.6 | 90.3 | **96.0** | | 85.4 | 86.1 | 81.9 |
| SDZB-15 | 83.3 | 90.9 | 95.7 | 90.6 | **96.1** | | 85.4 | 86.1 | 81.9 |
| SDZB2-15 | 84.0 | 91.5 | 95.9 | 91.0 | **96.2** | | 86.1 | 86.8 | 82.5 |
| Identity | 83.1-84.2 | 90.5-92.0 | 94.2-96.3 | 90.3-92.1 | **93.7-97.5** | | 85.3-86.7 | 86.0-87.4 | 81.7-82.5 |

The highest identity is identified in **Bold**.
