# Supplementary material for: Genetic Diversity and Positive Selection Analysis of Classical Swine Fever Virus Envelope Protein Gene E2 in East China under C-Strain Vaccination
Source: Front Microbiol. 2016 Feb 5;7:85. doi: 10.3389/fmicb.2016.00085 (PMC4742907; doi:10.3389/fmicb.2016.00085)
Supplement: Supplementary file 3 [file Table_3.DOC]

**Supplementary Table 3** Analysis of amino acid (AA) identity of E2 gene between 25 new isolates and other 8 representative CSFV isolates (%).

| New isolates | Shimen  (1.1) | SXCDK  (2.1a) | HEBZ  (2.1b) | GDPY2008  (2.1c) | | SDQS  (2.1d) | LAL290  (2.2) | Novska  (2.3) | TWN  (3.4) |
| --- | --- | --- | --- | --- | --- | --- | --- | --- | --- |
| SD19-15 | 88.7 | 95.2 | **96.2** | 95.4 | 95.7 | | 90.6 | 92.5 | 89.3 |
| SDJNi6-15 | 90.1 | 96.0 | **97.1** | 96.8 | 96.2 | | 92.0 | 93.6 | 89.8 |
| SDHZ-15 | 89.9 | 93.3 | 95.4 | 94.1 | **96.2** | | 89.5 | 91.2 | 88.5 |
| SDJNa-14 | 90.6 | 94.9 | 97.3 | 96.0 | **98.1** | | 91.4 | 92.8 | 89.8 |
| SDJNi1-15 | 90.3 | 94.6 | 97.1 | 95.7 | **97.9** | | 91.2 | 92.5 | 89.8 |
| SDJNi2-15 | 89.5 | 93.8 | 96.0 | 94.6 | **96.8** | | 90.1 | 91.4 | 89.0 |
| SDJNi3-15 | 89.8 | 94.4 | 96.8 | 95.4 | **98.1** | | 91.4 | 92.5 | 89.5 |
| SDJNi4-15 | 90.3 | 94.6 | 97.1 | 95.7 | **97.9** | | 91.2 | 92.5 | 89.5 |
| SDJNi5-15 | 90.6 | 94.9 | 97.3 | 96.0 | **98.1** | | 91.4 | 92.8 | 89.8 |
| SDLW1-15 | 89.5 | 94.1 | 96.5 | 95.2 | **97.9** | | 91.2 | 92.2 | 89.3 |
| SDLW2-15 | 90.9 | 95.2 | 97.6 | 96.2 | **98.4** | | 91.7 | 93.0 | 90.1 |
| SDLY-14 | 89.5 | 93.8 | 96.0 | 94.6 | **96.8** | | 90.1 | 91.4 | 89.0 |
| SDLY-15 | 89.5 | 93.8 | 96.2 | 94.9 | **97.6** | | 90.3 | 91.7 | 88.7 |
| SDMZ1-15 | 89.8 | 94.1 | 96.2 | 94.9 | **97.1** | | 90.3 | 91.7 | 89.3 |
| SDMZ2-15 | 90.1 | 94.4 | 96.8 | 95.4 | **97.6** | | 90.9 | 92.2 | 89.5 |
| SDSK-15 | 89.3 | 93.6 | 95.7 | 94.4 | **96.5** | | 89.8 | 91.2 | 88.7 |
| SDTA1-13 | 89.5 | 93.8 | 96.2 | 94.9 | **97.6** | | 90.3 | 91.7 | 88.7 |
| SDTA2-15 | 90.3 | 94.6 | 97.1 | 95.7 | **97.9** | | 91.2 | 92.5 | 89.5 |
| SDTA3-15 | 90.6 | 94.6 | 97.1 | 96.0 | **97.9** | | 91.2 | 93.0 | 89.8 |
| SDTA4-15 | 89.0 | 93.3 | 95.4 | 94.1 | **96.2** | | 89.5 | 90.9 | 88.5 |
| SDWK-15 | 89.8 | 94.1 | 96.5 | 95.2 | **97.3** | | 90.6 | 92.0 | 89.3 |
| SDXLS-15 | 89.8 | 94.1 | 96.2 | 94.9 | **97.1** | | 90.3 | 91.7 | 89.3 |
| SDXT-15 | 89.3 | 93.6 | 95.7 | 94.4 | **96.5** | | 89.8 | 91.2 | 88.7 |
| SDZB-15 | 89.5 | 93.8 | 96.0 | 94.6 | **96.8** | | 90.1 | 91.4 | 89.0 |
| SDZB2-15 | 90.6 | 94.9 | 97.3 | 96.0 | **97.6** | | 91.4 | 92.8 | 89.8 |
| Identity | 88.7-90.9 | 93.3-96.0 | 95.4-97.6 | 94.1-96.8 | **95.7-98.4** | | 89.5-92.0 | 90.9-93.6 | 88.5-90.1 |
